# Overexpression of VtF3′5′H and RhNHX Genes Alters Flower Color and Plant Morphology in Transgenic Rose ‘Red Farm’

**DOI:** 10.3390/plants14203185

**Published:** 2025-10-16

**Authors:** Ka Youn Lee, Su Young Lee, Yae Jin Kim, Youn Jung Choi, So Hyeon Lim, Yun-Im Kang

**Affiliations:** Floriculture Research Division, National Institute of Horticultural and Herbal Science, Rural Development Administration (RDA), Wanju 55365, Republic of Korea; kayoun200@korea.kr (K.Y.L.); lsy8542224@korea.kr (S.Y.L.); yj0503@korea.kr (Y.J.K.); lillium@korea.kr (Y.J.C.); hyeon9773@korea.kr (S.H.L.)

**Keywords:** rose (*Rosa hybrida*), flavonoid 3′,5′-hydroxylase, vacuolar sodium/proton antiporters, anthocyanin, gibberellin

## Abstract

Roses (*Rosa hybrida*) are among the most highly valued ornamental plants worldwide, with flower color serving as a major determinant of consumer preference and commercial success. However, the absence of the flavonoid 3′,5′-hydroxylase (*F3′5′H*) gene limits delphinidin biosynthesis, making it difficult to achieve blue or purple pigmentation. Vacuolar sodium/proton antiporters (*NHX*) regulate vacuolar pH and are also implicated in color stability. In this study, we introduced *Viola tricolor F3′5′H* (*VtF3′5′H*) and *Rosa hybrida NHX* (*RhNHX*) into the rose cultivar ‘Red Farm’ using Agrobacterium-mediated transformation. The non-native *VtF3′5′H* gene was detected in transgenic plants but not in the wild type, while *RhNHX* expression was relatively higher in transgenic plants. Petal anthocyanin content was significantly increased in T1–T4 compared to the wild type, and petal pH was also higher than that of the wild type. Growth and floral traits were also altered. Transgenic plants exhibited shorter stems, reduced stem diameter, more lateral branches, fewer prickles, and more than threefold higher petal numbers. Expression analysis showed reduced GA20-oxidase (*GA20ox1*) and GA3-oxidase (*GA3ox*) levels and increased GA2-oxidase (*GA2ox*) and GA2-oxidase6 (*GA2ox6*), particularly in stems, suggesting enhanced gibberellin (GA) inactivation. Overexpression of *VtF3′5′H* and *RhNHX* led to simultaneous changes in floral pigmentation and plant morphology. These findings indicate that both genes play functional roles in color development and growth regulation in roses.

## 1. Introduction

Flower color is a key trait of floricultural plants, contributing not only to aesthetics but also to economic value. From a consumer perspective, flower color is often the most important factor in purchasing decisions, with red and pink roses traditionally being the most popular choices [1,2]. However, Ellis and Ficek [3] report that men tend to prefer blue flowers, and female consumers aged 40–54 show a higher preference for blue-purple flowers than other age groups, indicating that color preferences vary with gender, age, and lifestyle [2]. Therefore, expanding the floriculture market requires the development of flowers in a wider range of colors that align with diverse consumer preferences.

Roses (*Rosa hybrida*) are among the most highly valued ornamental plants worldwide for their visual appeal and fragrance. Among rose characteristics, flower color has been a key factor in cultivar selection, as it directly affects consumer preference and commercial success. While researchers have previously developed diverse flower colors through traditional crossbreeding [4], recent efforts have focused on generating novel hues through genetic manipulation of the anthocyanin biosynthetic pathway [5,6].

Anthocyanins, metabolites of the flavonoid biosynthetic pathway, are major determinants of flower coloration [7,8,9]. They include cyanidin-3-glucoside (red), pelargonidin-3-glucoside (orange), and delphinidin-3-glucoside (blue/purple) [10]. Anthocyanin biosynthesis varies depending on the B-ring hydroxylation pattern, which is determined by flavonoid 3′-hydroxylase (*F3′H*) and flavonoid 3′,5′-hydroxylase (*F3′5′H*). An increase in the number of hydroxyl groups in this ring promotes delphinidin-based anthocyanin biosynthesis [11,12]. In particular, blue and purple pigmentation relies on delphinidin, for which *F3′5′H* is a key enzyme [13].

Several studies have demonstrated the central role of *F3′5′H*. In gentian cultivars Albireo, silencing of *F3′5′H* changed flower color from deep purple to light purple or light blue [14]. In *Hydrangea macrophylla*, suppression of *HmF3′5′H* using a TRV-*HmF3′5′H* construct resulted in a shift of sepal color from blue to pink, highlighting the importance of *F3′5′H* in delphinidin biosynthesis. However, plants such as Arabidopsis, roses, chrysanthemums, and carnations are unable to produce delphinidin-based pigments because they lack *F3′5′H* [15,16].

To overcome these limitations, researchers have attempted to generate blue pigmentation by introducing *F3′5′H*. Katsumoto et al. [13] successfully demonstrated delphinidin accumulation in rose petals up to 95% by introducing *F3′5′H* together with modification of the *DFR* substrate specificity. However, He et al. [17] reported that in chrysanthemum, suppression of *Chrysanthemum* × *morifolium* Ramat. *F3′H* and overexpression of *Senecio cruentus F3′5′H* alone did not enhance delphinidin accumulation, resulting instead in red flowers. These observations indicate that *F3′5′H* overexpression alone may not be sufficient for visible delphinidin pigmentation unless stabilization mechanisms such as vacuolar pH regulation are co-activated. Subsequently, Noda et al. [18] produced blue chrysanthemums by simultaneously introducing *Campanula medium F3′5′H* and *Clitoria ternatea A3′5′GT*, and Han et al. [19] showed that co-expression of *Osteospermum hybrid F3′5′H* and *Clitoria ternatea A3′5′GT* in the chrysanthemum ‘Nannong Fencui’ effectively converted pink flowers to purple or blue.

Flower coloration, however, is not determined solely by enzymes in the anthocyanin pathway. Factors such as vacuolar pH, metal ion complexation, and other pigments are also involved [20,21]. In particular, the visible spectrum of flavonoids is strongly pH-dependent, which explains why vacuolar pH plays a central role in color expression [22]. Anthocyanins exist as flavylium cations (red to purple) under low pH but shift to quinonoidal base forms at higher pH [23,24]. Roses, which typically have highly acidic vacuoles, often display red coloration even when delphinidin-type anthocyanins accumulate [13]. Therefore, artificial regulation of vacuolar pH is essential for achieving blue pigmentation in roses. *NHX* localize to the vacuolar membrane, where they regulate pH via cation exchange and modulate osmotic balance and cell expansion through K^+^ and Na^+^ transport, thus playing critical roles in growth and development [25,26,27,28]. Overexpression of *NHX* has been reported to enhance color intensity and stability [29,30]. Recently, Wang et al. [31] reported that overexpression of *RcNHX2* altered petal pH and induced bluish pigmentation, whereas silencing *RcNHX2* weakened flower coloration.

Taken together, these studies indicate that both anthocyanin biosynthesis and *NHX*-mediated vacuolar regulation must be considered to achieve stable and vivid flower colors in roses. In this study, we introduced *VtF3′5′H* and *RhNHX* into the rose ‘Red Farm’. We analyzed flower color, vacuolar pH, growth characteristics, and expression of GA biosynthetic genes in transgenic plants to explore the potential roles of Vt*F3′5′H* and *RhNHX* in modulating pigment accumulation and growth regulation.

## 2. Results

### 2.1. Confirmation of Gene Introduction in Transgenic Plants

**Figure 1:**
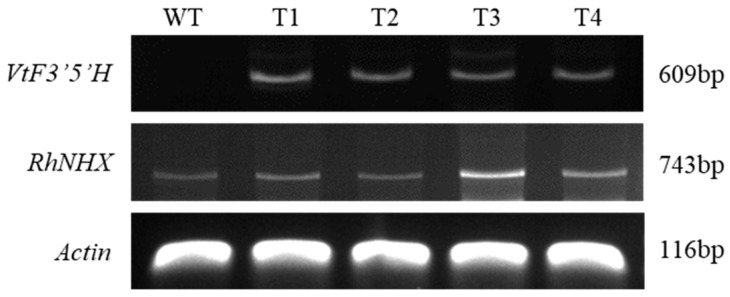
PCR confirmation of transgene integration in wild-type (WT) and transgenic ‘Red Farm’ rose lines (T1–T4). Amplification of *VtF3′5′H* (609 bp) and *RhNHX* (743 bp) was detected in transgenic lines but not in WT. Actin (116 bp) was used as the internal control.

As expected, the non-native *VtF3′5′H* gene showed no amplification in the wild type, whereas a clear 609 bp band was detected in transgenic plants T1–T4, confirming successful gene integration. In addition, Sanger sequencing of the PCR amplicons verified that the inserted fragments shared 99–100% identity with the original *VtF3′5′H* sequence (Figure S1), validating the integrity of the introduced transgene. The endogenous *RhNHX* gene (743 bp) was detected in both wild-type and transgenic plants, with relatively higher expression in the transgenic plant (Figure 1).

### 2.2. Anthocyanin Accumulation and Petal pH Changes

**Figure 2:**
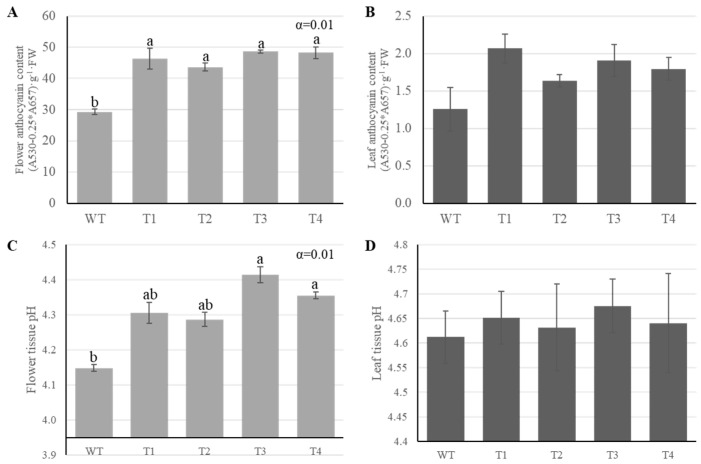
Anthocyanin content and pH measurements in wild type (WT) and transgenic ‘Red Farm’ rose lines (T1–T4). (**A**) Petal anthocyanin content was significantly increased in transgenic lines compared with WT (α = 0.01). (**B**) Leaf anthocyanin content showed no significant differences between WT and transgenic lines (ns). (**C**) Petal tissue pH was significantly elevated in transgenic lines compared with WT (α = 0.01). (**D**) Leaf tissue pH showed no significant differences between WT and transgenic lines (ns). Bars show mean ± SE (n = 3). Lowercase letters indicate multiple-comparison groupings from one-way ANOVA with Tukey’s HSD; bars sharing a letter are not significantly different. ns indicates no significant difference (α = 0.05).

Anthocyanin content was measured to assess pigment accumulation in leaves and flowers. Petal anthocyanin content increased significantly by ~50–65%, from 29.4 ± 0.8 in the wild type to 43.6–48.6 in T1–T4 (Figure 2C). The pH of petals was found to be higher in the transgenic plants (4.3–4.4) than in the wild type (4.1 ± 0.01), and this trend was consistent with the increased anthocyanin content observed in transgenic petals (Figure 2D). Meanwhile, the pH of the leaves showed no difference between the transgenic plants and the wild type.

### 2.3. Growth Trait Changes in Transgenic Plant

**Figure 3:**
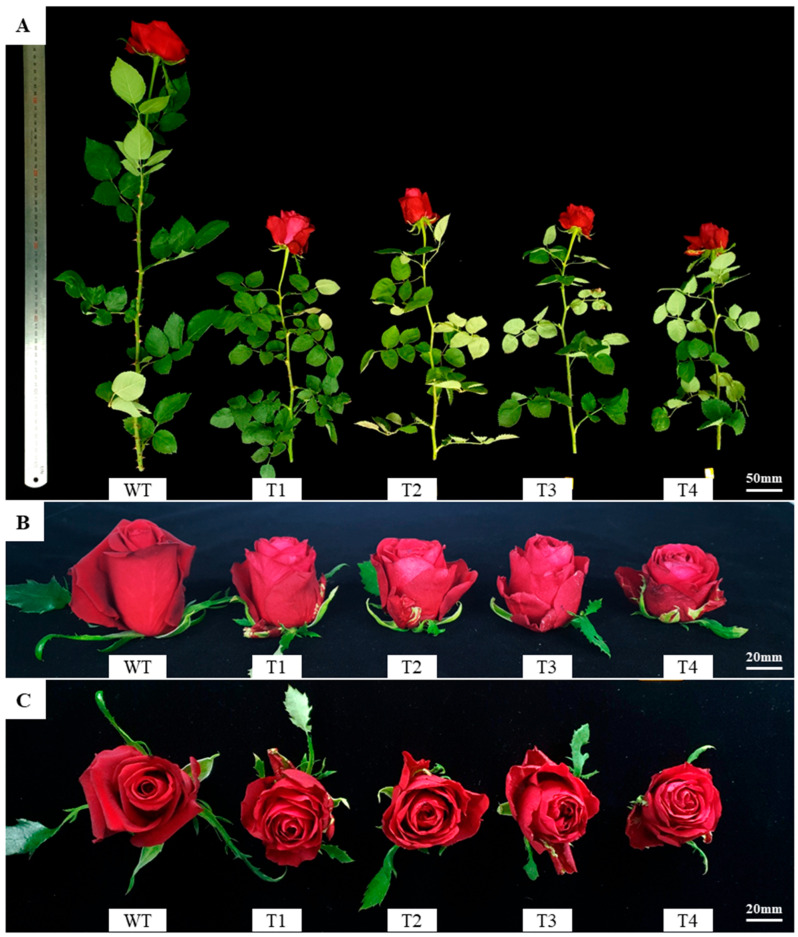
Phenotypic differences in growth and flower morphology between wild-type and transgenic lines. (**A**) Whole-plant morphology of wild type (WT) and transgenic lines (T1–T4) after soil acclimatization (Bar = 50 mm). (**B**) Side view of floral buds showing reduced flower height in transgenic lines compared with WT (Bar = 20 mm). (**C**) Top view of fully opened flowers showing reduced flower width but increased petal number, resulting in denser floral structures in the transgenic lines (Bar = 20 mm).

Comparison of growth and flowering characteristics between transformed and wild type revealed significant differences in several parameters. Stem length in T1–T4 was reduced by ~45–55%, ranging from 29.5 to 36.7 cm, compared with 66.8 cm in the wild type (Figure 3A, Table 1). Stem diameter was also significantly smaller in T2 and T4 (2.9 mm) compared to the wild type (4.2 mm). These results show that stem elongation and thickening were suppressed in the transgenic plant.

Peduncle length and diameter were likewise significantly lower than those of the wild type. In contrast, the number of lateral branches increased more than fivefold, from 0.5 in the wild type to 2.8–4.4 in the transgenic plant. The number of prickles was also significantly fewer, averaging 13.6 in the wild type but only 0.8–2.8 in the transgenic plant. The number of leaves also tended to decrease in the transgenic plant (5.8–6.9) compared with the wild type (8.0). Accordingly, fresh weight was significantly reduced, with T4 averaging 11.1 g, approximately half that of the wild type (22.6 g). Notably, floral traits, both flower diameter and height, were smaller in the transgenic plant compared with the wild type (Figure 3B,C). However, petal number increased more than threefold, from 27.8 in the wild type to 75.9–87.9 in the transgenic plant.

### 2.4. Expression of Genes Related to GA Metabolism

**Figure 4:**
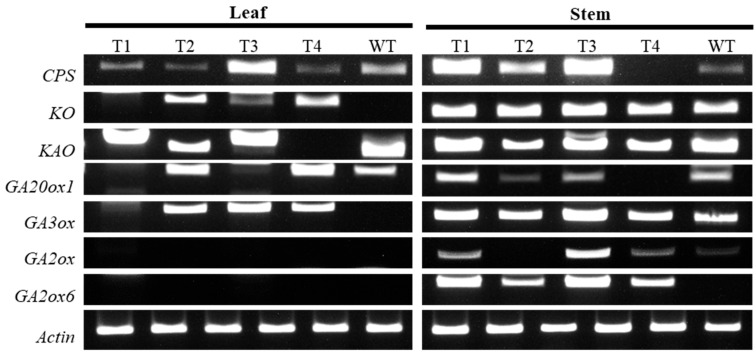
Expression analysis of gibberellin biosynthesis–related genes in leaves and stems of wild type (WT) and transgenic ‘Red Farm’ rose lines (T1–T4). RT-PCR was performed for *CPS*, *KO*, *KAO*, *GA20ox1*, *GA3ox*, *GA2ox*, and *GA2ox6*, with Actin used as the internal control.

To investigate the cause of suppressed internode elongation in the transgenic plant, we analyzed the expression of seven genes involved in gibberellin biosynthesis in leaves and stems: ent-copalyl diphosphate synthase (*CPS*), ent-kaurene oxidase (*KO*), ent-kaurenoic acid oxidase (*KAO*), GA20-oxidase (*GA20ox1*), GA3-oxidase (*GA3ox*), GA2-oxidase (*GA2ox*), and GA2-oxidase6 (*GA2ox6*). The expression levels of *GA20ox1* and *GA3ox*, two downstream genes involved in active gibberellin biosynthesis, were relatively reduced compared with the wild type (Figure 4). In contrast, the expression levels of *GA2ox* and *GA2ox6*, which inhibit active gibberellin biosynthesis, were increased in the transgenic plant. In particular, *GA2ox* expression was higher in stems than in leaves of the transgenic plant, suggesting that gibberellin inactivation was more pronounced in stem tissues.

## 3. Discussion

In this study, a ‘Red Farm’ rose transgenic plant co-expressing *VtF3′5′H* and *RhNHX* was generated. Anthocyanin content was significantly increased in the petals of the transgenic plant compared with the wild type, accompanied by a significant increase in petal pH. However, our measurements represent tissue-level averages, with vacuolar pH likely contributing the most because the vacuole is the largest organelle in plant cells. Previous studies have demonstrated that flavonoid absorption spectra are strongly pH-dependent [22], supporting the interpretation that NHX-mediated pH modulation can alter pigment stabilization. Furthermore, vacuolar pH regulation may interact with local hormonal balance and membrane transport processes (e.g., V-ATPase, V-PPase), which could partly explain the growth-related phenotypes observed in the transgenic lines. Future studies using vacuole-targeted pH probes in epidermal cells, where anthocyanins primarily accumulate, will be required for more accurate pH analysis. Such studies could provide more biologically meaningful insights into the role of *NHX* in vacuolar pH regulation and pigment stabilization. Therefore, the results of this study should be interpreted as tissue-level estimates for comparing relative differences between control and transgenic plants. Fukada-Tanaka et al. [24] reported that an *InNHX1* recessive mutant in *Ipomoea tricolor* maintained a low vacuolar pH, causing blue (wild type) flowers to appear purple. In Phalaenopsis, downregulation of *PeNHX1* reduced petal pigmentation and increased vacuolar pH, whereas its overexpression induced a color shift from red to blue [32]. These findings support the possibility that the flower color change in the ‘Red Farm’ transgenic plant may be associated with delphinidin synthesis induced by *VtF3′5′H*, together with *NHX*-mediated regulation of vacuolar pH, which could contribute to pigment accumulation and stabilization. Xu et al. [33] further confirmed that co-introduction of *VtF3′5′H* and *RhNHX* in roses altered flower color from white to pink. Our results are in agreement with these previous studies and suggest that the two genes may function together to influence anthocyanin accumulation and stabilization.

Several factors, including temperature, oxygen, and metal ions, influence the stability of anthocyanins; in particular, stability is highly dependent on pH [34,35]. Stavenga et al. [22] showed spectroscopically that the absorbance spectrum of anthocyanin solutions shifted from violet to blue as pH increased from acidic to neutral or alkaline. In this study, petals of transgenic plants showed significantly higher pH than those of the wild type, whereas no significant difference was detected in leaves, indicating that expression levels may vary among tissues even when driven by a constitutive promoter. Therefore, vacuolar pH is a key determinant of flower color, given that anthocyanins are primarily localized in the vacuoles of epidermal cells. NHX proteins maintain K^+^ and Na^+^ homeostasis, regulate vacuolar pH, and influence osmotic balance, cell expansion, and development, thereby contributing to diverse physiological processes [27,29,36]. In *Arabidopsis*, the *NHX1 NHX2* double mutant exhibited not only growth retardation (dwarf phenotype) and impaired cell expansion, but also reproductive defects, including seedlessness and short, narrow flower organs [30]. In this study, transgenic plants displayed suppressed internode elongation, reduced plant height, decreased number of prickles, and smaller flower width and height compared with the wild type. In this transgenic plant, petal pH was higher than in the wild type, consistent with previous reports showing that vacuolar alkalization is required to stabilize delphinidin-based pigments. These results raise the possibility that *NHX* may contribute to both pigment stabilization and growth-related processes.

Because reduced internode length and altered shoot architecture were consistently observed, GA metabolism was examined as a supplementary approach to aid the interpretation of these phenotypic changes. In transgenic stems, *GA2ox* was higher, and *GA20ox* and *GA3ox* expression were lower compared with the wild type, which is consistent with the phenotypic data. These findings align with previous reports that *GA2ox* overexpression induces a dwarf phenotype [37,38]. Moreover, GA signaling regulates stem elongation through crosstalk with other hormonal pathways such as auxin and brassinosteroids [39,40,41,42,43,44]. Although the present findings do not establish a direct mechanistic link, these observations raise the possibility that *RhNHX* expression may indirectly influence GA-associated growth responses. Further investigation will be required to clarify whether these transcriptional changes arise from ionic effects or hormonal crosstalk.

Meanwhile, the transgenic plants showed a statistically significant increase in the number of lateral branches compared with the wild type. The increase in lateral branches may enhance flowering capacity, providing potential benefits for floriculture production. Another notable change was a significant reduction in the number of prickles, which could improve cut flower quality and ease of handling for growers [45,46]. Furthermore, the shorter plant stature could be advantageous for certain markets, such as potted or miniature roses. Therefore, co-introducing *VtF3′5′H* and *RhNHX* represents a potential strategy not only to alter flower color but also to enhance the commercial value of roses.

Among the various cellular compartments, vacuolar pH is considered the most variable and biologically relevant factor. Its modulation can directly influence pigment stabilization [25,47] and may also interact with hormone transport pathways, thereby contributing to the growth phenotypes observed in the transgenic plants. Consistent with our observations, Bassil et al. [30] demonstrated that *NHX*-mediated vacuolar ion homeostasis is required for cell expansion and organ development in *Arabidopsis*, further supporting the role of vacuolar pH as a key regulator linking both flower color stabilization and plant architecture.

In this study, we analyzed the expression of genes involved in gibberellin biosynthesis in relation to growth using semi-quantitative RT-PCR. Similar insertional effects have been reported in other Agrobacterium-mediated transformation studies, where T-DNA integration unexpectedly altered plant architecture independently of the target gene function [48]. While such insertion-induced effects cannot be completely excluded in our lines, the consistent correlation between morphological and biochemical changes strongly support a role for overexpression of *VtF3′5′H* and *RhNHX* in the observed phenotypes. The results suggest that *NHX* may affect hormone signaling. To more clearly distinguish these effects, future analyses using qRT-PCR and LC-MS will provide stronger evidence to support this hypothesis.

Stewart et al. [49] highlighted the commercial importance of dwarf or compact varieties in the ornamental plant industry, noting their advantages in management efficiency and market applicability. Therefore, the shorter internode length observed in our transgenic lines should not necessarily be interpreted as a detrimental effect but rather as a potentially valuable horticultural trait. Taken together, these findings indicate that *RhNHX* and *VtF3′5′H* influence both flower color and morphological traits in roses, providing new insights into the functional link between NHX and GA metabolism. These results demonstrate the potential to simultaneously improve flower color and horticultural performance and provide a foundation for future breeding strategies.

## 4. Materials and Methods

### 4.1. Plant Materials and Culture Conditions

**Figure 5:**
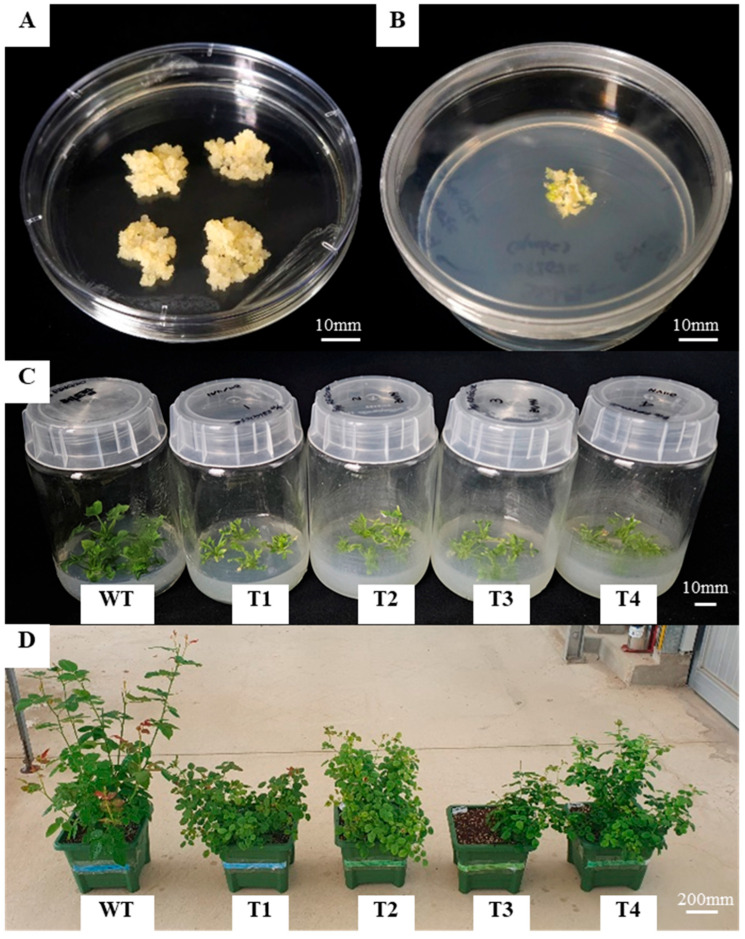
Generation and growth of transgenic ‘Red Farm’ roses harboring *RhNHX* and *VtF3′5′H*. (**A**) Embryogenic callus induced from root-derived explants of ‘Red Farm’ rose (Bar = 10 mm). (**B**) Callus proliferation after Agrobacterium inoculation and co-cultivation, grown on selection medium (Bar = 10 mm). (**C**) Regenerated shoots of wild type (WT) and transgenic lines (T1–T4) (Bar = 10 mm). (**D**) Growth performance of WT and transgenic plants after acclimatization in soil (Bar = 200 mm).

In this study, embryogenic callus derived from in vitro-cultured roots of the rose ‘Red Farm’ (bred at the National Institute of Horticultural & Herbal Science; NIHHS) was used for transformation (Figure 5A). To induce embryogenic callus, roots were plated on Schenk & Hildebrandt (SH) medium [50] supplemented with 5 or 11 mg·L^−1^ 2,4-D and cultured for 8 weeks. The induced callus was subcultured to SH medium supplemented with 3 mg·L^−1^ 2,4-D, 300 mg·L^−1^ L-proline, 30 g·L^−1^ sucrose, and 2.4 g·L^−1^ phytagel. The calli were maintained on the same medium at 4–6-week intervals at 25 ± 5 °C under a 16 h light/8 h dark photoperiod for propagation.

### 4.2. Preparation of Transformation Plasmids

The *F3′5′H* gene, involved in blue pigment biosynthesis, was isolated from petals of pansy line PS-12-34, bred at the Korea National University of Agriculture and Fisheries. The *NHX* gene, which influences flower color, was obtained from rose line 10R-40-31, bred at the NIHHS. Total RNA was extracted from the petals of each plant using the RNeasy Plus Mini Kit (Qiagen, Hilden, Germany), and cDNA was synthesized using the PrimeScript 1st Strand cDNA Synthesis Kit (TaKaRa, Madison, WI, USA). PCR was performed using synthesized cDNA and Ex-Taq DNA polymerase (TaKaRa, Madison, WI, USA). PCR conditions were: initial denaturation at 95 °C for 30 s; 35 cycles of 95 °C for 30 s, 58 °C for 30 s, and 72 °C for 30 s. The PCR products were ligated into pGEM-T Easy Vector (Promega, Madison, WI, USA) and transformed into *E. coli* DH5α (TaKaRa, Madison, WI, USA). Plasmids were extracted from selected colonies using the QIAprep Spin Miniprep Kit (Qiagen, Hilden, Germany), and the presence of the genes was confirmed by sequence analysis.

The *Agrobacterium tumefaciens* strain LBA4404, harboring the pPZP200 vector containing *RhNHX* and *VtF3′5′H*, was used in this study. The *RhNHX* and *VtF3′5′H* genes were expressed by the cauliflower mosaic virus 35S (CaMV35S) promoter. Phosphinothricin (PPT) resistance was used as a selection marker. The complete plasmid sequences, including the full-length constructs and vector maps used for transformation, are provided in Figure S2.

### 4.3. Agrobacterium Cultivation and Co-Culture

Transformation was performed by the modified method described by Lee et al. [51,52,53]. Transformed Agrobacterium was cultured in LB medium at 28 °C and 200 rpm for 15 h and then inoculated with embryogenic callus at 100 rpm for 30 min. Calli were transferred to SH medium supplemented with 1 mg·L^−1^ of BA, 0.1 mg·L^−1^ of IBA, 30 g·L^−1^ of maltose, 250 mg·L^−1^ of cefotaxime, and 4 g·L^−1^ of agarose and co-cultured in the dark for 3 days. Afterward, calli were transferred to selection medium containing 2 mg·L^−1^ PPT and subcultured every 4 weeks to obtain adventitious shoots (Figure 5B,C).

### 4.4. PCR Confirmation and Acclimatization of Transgenic Plants

After acclimating plants with healthy shoots and roots to the soil, they were transferred to the greenhouse for growth (Figure 5D). To confirm gene introduction, leaves were rapidly frozen in liquid nitrogen and finely ground using a mortar and pestle. DNA was extracted using the DNeasy Plant Mini Kit (Qiagen, Hilden, Germany) and amplified with Ex-Taq DNA polymerase (TaKaRa, Madison, WI, USA). Gene-specific primers for *VtF3′5′H* and *RhNHX* were used for introduction (Table S1). PCR conditions were 95 °C for 5 min, followed by 35 cycles of 94 °C for 30 s, 57 °C for 30 s, and 72 °C for 1 min, and final extension at 72 °C for 5 min. pPZP200 vector containing the *VtF3′5′H* and *RhNHX* genes was used as a positive control. PCR products were separated on a 1.5% agarose gel at 100 V for 70 min.

### 4.5. Analysis of Total Anthocyanin and pH

All plants were cultivated in a glass greenhouse under natural sunlight at approximately 25–28 °C, using a commercial horticultural substrate mix (Baroker; Seoul Bio, Eumseong-gun, Republic of Korea). Anthocyanin content of greenhouse-grown transgenic plants was measured as described previously [54]. Fully opened petals and young leaves were collected, frozen in liquid nitrogen, and ground. A 0.15 g sample was homogenized with 1.5 mL of extraction solution (99:1 *v*/*v* methanol/HCl; Sigma-Aldrich, St. Louis, MO, USA). Extracts were kept in darkness at 4 °C for 24 h, then centrifuged at 13,000 rpm for 20 min at 4 °C. Absorbance was recorded at 530 and 657 nm using a QuickDrop spectrophotometer (Molecular Devices, San Jose, CA, USA). Anthocyanin content was calculated as (A530 − 0.25 × A657)/fresh weight.

Petal and leaf pH were measured following Manteau et al. [55]. Approximately 1 g of petal or young leaf tissue was homogenized in distilled water, and the resulting tissue suspension was immediately measured using a pH meter. These measurements represent tissue-level average pH values of homogenized petal and leaf tissues and were used for relative comparisons between transgenic and control plants, rather than for organelle- or cell-type–specific interpretations.

### 4.6. Phenotypic Characteristics of Transgenic Plants

Phenotypic traits of transgenic plants and the wild type were assessed at flowering. Flower color was evaluated with the RHS Color Chart (Royal Horticultural Society, London, UK). Growth and floral traits were investigated according to the guidelines for evaluating new rose varieties. Overall growth traits (cut flower length, stem diameter, number of leaves, fresh weight, number of lateral branches, number of prickles) and floral traits (peduncle length, peduncle diameter, petal number, flower width, flower height) were recorded.

### 4.7. Gene Expression Analysis of the Gibberellin Biosynthesis Pathway

Because transgenic plants exhibited altered stem elongation, expression of seven gibberellin biosynthetic genes (*CPS*, *KO*, *KAO*, *GA20ox1*, *GA3ox*, *GA2ox*, *GA2ox6*) was analyzed. For gene expression analysis, leaves and stems at the first internode from the shoot tips were collected from four transformant lines and the wild type. Samples were frozen in liquid nitrogen and powdered using a mortar and pestle. RNA was extracted using Fruit-Mate for RNA Purification (TaKaRa, Madison, WI, USA) and the RNeasy Plant Mini Kit (Qiagen, Hilden, Germany). RNA was synthesized into cDNA using the PrimeScript RT Reagent Kit with gDNA Eraser (TaKaRa, Madison, WI, USA). PCR was performed using EmeraldAmp GT PCR Master Mix (TaKaRa, Madison, WI, USA) under the following conditions: 94 °C for 1 min (denaturation), 57 °C for 1 min (annealing), 72 °C for 1 min (extension), for 35 cycles. Primers were designed based on sequences in NCBI (National Center for Biotechnology Information) (Table S1).

### 4.8. Statistical Analysis

Data were analyzed with one-way ANOVA using SPSS 20.0 (IBM Corp., Armonk, NY, USA). Duncan’s multiple range test (DMRT) was applied for post hoc comparisons. Differences were considered significant at *p* ≤ 0.05.

## 5. Conclusions

In this study, co-introduction of *Viola tricolor F3′5′H* and *Rosa hybrida NHX* into the rose cultivar ‘Red Farm’ enhanced anthocyanin accumulation in petals and altered vacuolar pH. Significant modifications in growth and flowering traits were also observed, including increased petal anthocyanin content, reduced stem elongation and thickness, enhanced branching, and fewer prickles. Expression analysis further revealed up-regulation of *GA2ox* and *GA2ox6* in stems, indicating enhanced gibberellin inactivation.

The observed changes raise the possibility that *NHX* affects not only vacuolar pH and pigment stabilization but may also be indirectly associated with growth regulation. Future quantitative gene expression and metabolite analyses will provide strong foundational data to support the hypothesis that *NHX* contributes to both pigment stabilization and hormone signaling. Ultimately, this study highlights a breeding strategy for developing rose cultivars with enhanced ornamental and horticultural value by broadening flower color diversity through the regulation of anthocyanin biosynthesis and vacuolar ion transport.

## Figures and Tables

**Table 1:** Growth and floral characteristics of wild-type and *RhNHX*/*VtF3′5′H* transgenic lines (T1–T4) of ‘Red Farm’ roses.

	Stem length (cm)			Stem diameter(mm)				No. of leaves		No. of prickles				No. of branches				Fresh weight (g)		
Wild type	66.8	a		4.2		a		8	a	13.6		a		0.5		b		22.6		a
T1	32.7	b		3.3		ab		6	ab	2.1		b		4		ab		17.6		ab
T2	31	b		2.9		b		5.8	b	2.8		b		2.8		ab		14.9		ab
T3	36.7	b		3.1		b		6.9	ab	2.4		b		3.4		ab		15.2		ab
T4	29.5	b		2.9		b		6.9	ab	0.8		b		4.4		a		11.1		b
Significance ^z^	***			*				*		***				***				*		
	Peduncle length (cm)				Peduncle diameter (mm)				No. of petals				Flower width (cm)				Flower height (cm)			
Wild type	13.6		a		3.1		a		27.8		b		6.4		a		4.8		a	
T1	8.7		b		2.3		ab		87.9		a		5.5		ab		4.4		ab	
T2	8.1		b		2.1		b		75.9		a		5.4		ab		4.1		abc	
T3	7.2		b		2.3		ab		78		a		5		b		3.7		bc	
T4	7.7		b		1.8		b		79.4		a		4.9		b		3.4		c	
Significance ^z^	***				***				***				*				***			

^z^ Means followed by the same letter are not significantly different from each other at (α = 0.001 (***), 0.05 (*)) determined using Duncan’s multiple range test (DMRT).

## Data Availability

The original contributions presented in this study are included in the article/Supplementary Materials. Further inquiries can be directed to the corresponding author.

## Supplementary Materials

The following supporting information can be downloaded at: https://www.mdpi.com/article/10.3390/plants14203185/s1, Figure S1: Alignment of PCR-amplified *VtF3′5′H* fragments with the original vector sequence. Figure S2: Complete plasmid sequences and corresponding vector maps used for transformation. Table S1: Primer sequences used for amplification and transgene detection.
