# Supplementary material for: Overexpression of VtF3′5′H and RhNHX Genes Alters Flower Color and Plant Morphology in Transgenic Rose ‘Red Farm’
Source: Plants (Basel). 2025 Oct 16;14(20):3185. doi: 10.3390/plants14203185 (PMC12567232; doi:10.3390/plants14203185)
Supplement: Supplementary file 1 [file plants-14-03185-s001.zip › plants-3903203-supplementary.pdf]

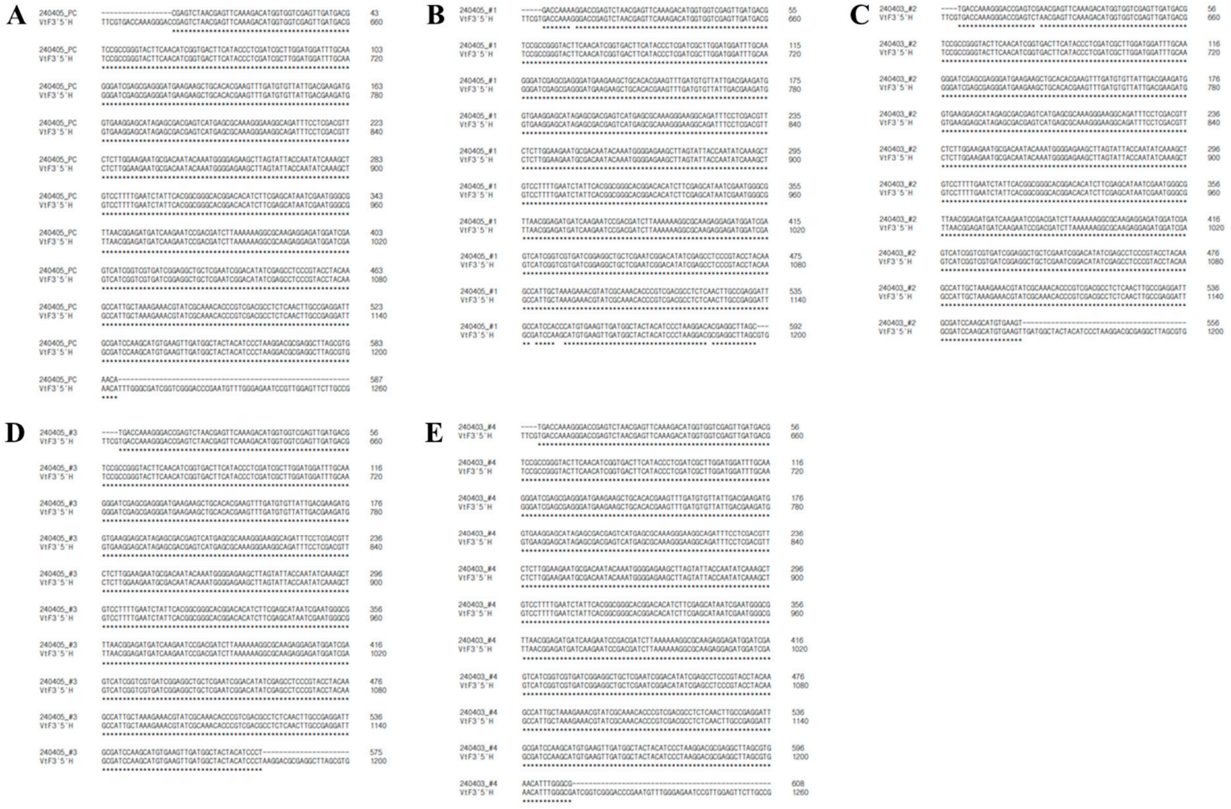

**Figure S1.** Alignment of PCR-amplified VtF3′5′H fragments with the original vector sequence. (A) Positive control (PC) showing sequence identity with the reference construct. (B–E) Sequencing results from transgenic lines T1–T4 aligned to the *VtF3′5′H* sequence in the transformation vector, confirming 99–100% similarity.

- Vector map of **pPZP-NHX9-Vt-F35H**

**RB-P35S-NHX9-PINII-P35S-Vt_F35H-T35S-Bar—3'NOS-LB-Backbone**

- Sequence of **pPZP-NHX9-Vt_F35H**

CGCGTGCAGTTCGGCCCGTTGGTTGGTCAAGTCCTGGTCGTCGGTGCTGACGCGGGCATAGCCCAGCAGGCCAGCGG
CGGCGCTCTTGTTCATGGCGTAATGTCTCCGGTTCTAGTCGCAAGTATTCTACTTTATGCGACTAAAACACGCGACA
AGAAAACGCCAGGAAAAGGGCAGGGCGGCAGCCTGTCGCGTAACTTAGGACTTGTGCGACATGTCGTTTTCAGAAGA
CGGCTGCACTGAACGTCAGAAGCCGACTGCACTATAGCAGCGGAGGGGTTGGATCAAAGTACTTTAAAGTACTTTGA
TCCCGAGGGGAACCCTGTGGTTGGCATGCACATACAAATGGACGAACGGATAAACCTTTTCACGCCCTTTTAAATAT
CCGATTATTCTAATAAACGCTCTTTTCTCTTAGGTTTACCCGCCAATATATCCTGTCAAACACTGATAGTTTAAACT
GAAGGCGGGAAACGACAATCTGATCCAAGCTCAAGCTAAGCTTGCATGCCTCGAGGTCCCCAGATTAGCCTTTTCAA
TTTCAGAAAGAATGCTAACCCACAGATGGTTAGAGAGGCTTACGCAGCAGGTCTCATCAAGACGATCTACCCGAGCA
ATAATCTCCAGGAAATCAAATACCTTCCCAAGAAGGTTAAAGATGCAGTCAAAAGATTCAGGACTAACTGCATCAAG
AACACAGAGAAAGATATATTTCTCAAGATCAGAAGTACTATTCCAGTATGGACGATTCAAGGCTTGCTTCACAAACC
AAGGCAAGTAATAGAGATTGGAGTCTCTAAAAAGGTAGTTCCCACTGAATCAAAGGCCATGGAGTCAAAGATTCAAA
TAGAGGACCTAACAGAACTCGCCGTAAAGACTGGCGAACAGTTCATACAGAGTCTCTTACGACTCAATGACAAGAAG
AAAATCTTCGTCAACATGGTGGAGCACGACACACTTGTCTACTCCAAAAATATCAAAGATACAGTCTCAGAAGACCA
AAGGGCAATTGAGACTTTTCAACAAAGGGTAATATCCGGAAACCTCCTCGGATTCCATTGCCCAGCTATCTGTCACT
TTATTGTGAAGATAGTGGAAAAGGAAGGTGGCTCCTACAAATGCCATCATTGCGATAAAGGAAAGGCCATCGTTGAA
GATGCCTCTGCCGACAGTGGTCCCAAAGATGGACCCCCACCCACGAGGAGCATCGTGGAAAAAGAAGACGTTCCAAC
CACGTCTTCAAAGCAAGTGGATTGATGTGATATCTCCACTGACGTAAGGGATGACGCACAATCCCACTATCCTTCGC
AAGACCCTTCCTCTATATAAGGAAGTTCATTTCATTTGGAGAGAACACGGGGGACTCTACTGCAGGAATTCGATTTG
GTTGTGTTGAAGACGAGGATACAATGGCTTCTCATTTGGCCATGTTGATGACCAAGTTACCCAAATTGCAAAATCTA
TCTGCTTCTGATCACTCCTCTGTTGTCTCGTTGAACCTTTTCGTGGCACTACTTTGTGCTTGTATTGTGATTGGGCA
TCTTCTTGAGGAGAATCGGTGGGTGAACGAGTCAATCACCGCCCTTTTGATTGGGGTGTCTACTGGAGTACTTATTC
TTCTGATCAGTGGAGGAAAAAGTTCGCATCTTTTAGTATTCAGTGAAGATCTTTTCTTTATTTACCTTCTACCACCT
ATTATTTTTAATGCCGGGTTTCAGGTGAAAAAGAAGCAGTTTTTCCGCAATTTCATTACTATTGTAATGTTCGGTGC
TATTGGTACATTAGTATCCTGCACCATTATAGCATTTGGTGCTACACAAATCTTTAAGAAATTGGACATTGGTTCGC
TGGACATAGGGGATTATCTCGCAATTGGTGCAATATTTGCTGCAACGGATTCTGTATGCACGTTGCAGGTGCTCCAT
CAGGATGAGACTCCTTTACTGTACAGTCTTGTATTCGGCGAGGGAGTTGTTGATGATGCTACATCTGTGGTGCTTTT
CAATGCTATCCAGAGCTTTGATCTAACACACCTTGATTCCAGAATCGCCTTGAAGTTTATGGGCAACTTTTTGTATT
TGTTTTTTGCAAGCACCATGCTAGGAGTGATTACAGGGCTGCTAAGTGCTTTCATTATCAAAAAGCTTTATTTTGCA
AGGCACTCAACAGATCGTGAGGTCGCTCTTATGATGCTCATGGCATACCTTTCATATATACTGGCTGAACTATTCTA
TTTGAGTGGCATTCTCACTGTATTCTTTTGTGGGATTGTGATGTCCCATTACACCTGGCACAATGTGACAGAGAGTT
CAAGGGTCACGACCAAGCATGCTTTTGCAACCTTGTCATTTGTTTGCGAGACTTTTATCTTCCTCTATGTTGGTATG
GATGCCTTGGACATTGAAAAGTGGAGATTTGTAAGTGATAGTCCTGGAACGTCAGTGGCAGTGAGTTCAATACTGCT
AAGTCTTGTTATGCTTGGAAGAGCAGCTTTTGTTTTTCCTTTATCCTTTTTCTCAAACTTATTTAAGAAAAATCAAA
GCGAGAAAATTAGCCTCCAGCAGCAAGTGGTAATATGGTGGGCTGGTCTTATGAGAGGTGCTGTGTCTATAGCGCTT
GCTTATAATCAGTTTACAAGATCTGGTCACACTCAATTGCGAGCAAATGCAATCATGATCACTAGCACAATAAGTGT
TGTTCTTGTCAGCACAGTGGTGTTTGGTTTGATGACGAAACCTCTTATTAGATTATTGCTGCCTCATAAACAATTGA
CCAGCACAAACAGCATTATGTCAGACCCACCCTCTCCAAAATCAGTCATTGTTCCACTTCTTGGGCAGGATTCTGAA
GCTGATCTGAGCGGTCATGAGGTGCGTCGTCCAGCCAGCATACGTGATCTTCTGACGACTCCAACACACACTGTACA
TCGCTACTGGCGTAAGTTTGATAATGCTTTCATGCGTCCAGTATTTGGTGGTCGGGGTTTTGTTCCCTTTGTTCCCG
GCTCACCAACTGAACGGAACAACACTCAATGGCAATGAGTCAAGCAAAATGTGAAAAAAGTTTGTAAGTCATGTATA
ATATCTAGAAATGCAGGACTTCAATTCAGCGTGTGATTATTTTACGAAATCACTAGTGAATTCGATGGGACCCTGCA
ATGTGACCCTAGACTTGTCCATCTTCTGGATTGGCCAACTTAATTAATGTATGAAATAAAAGGATGCACACATAGTG
ACATGCTAATCACTATAATGTGGGCATCAAAGTTGTGTGTTATGTGTAATTACTAATTATCTGAATAAGAGAAAGAG
ATCATCCATATTTCTTATCCTAAATGAATGTCACGTGTCTTTATAATTCTTTGATGAACCAGATGCATTTTATTAAC
CAATTCCATATACATATAAATATTAATCATATATAATTAATATCAATTGGGTTAGCAAAACAAATCTAGTCTAGGTG
TGTTTTGCTAATTATTGGGGGATAGTGCAAAAAGAAATCTACGTTCTCAATAATTCAGATAGAAAACTTAATAAAGT
GAGATAATTTACATAGATTGCTTTTATCCTTTGATATATGTGAAACCATGCATGATATAAGGAAAATAGATAGAGAA
ATAATTTTTTACATCGTTGAATATGTAAACAATTTAATTCAAGAAGCTAGGAATATAAATATTGAGGAGTTTATGAT
TATTATTATTATTTTGATGTTCAATGAAGTTTTTTTTAATTTCATATGAAGTATACAAAAATTCTTCATAGATTTTT
GTTTCTATGCCGTAGTTATCTTTAATATATTTGTGGTTGAAGAAATTTATTGCTAGAAACGAATGGATTGTCAATTT

TTTTTTAAAGCAAATATATATGAAATTATACTGTATATTATTTTAGTCATGATTAAAATGTGGCCTTAATTGAATCA
TCTTTCTCATTCATTTTTTC**A**AAAGCATATCAGGATGATTGATATTTATCTATTTTAAAA**A**TTAATTTAA**G**GGTTCA
AATTAAATTTAACTTAAAAGTGTCCTAACCGTAGTTAAAGGTTTACTTTAAAAAAATACTATGAAAAATCTAATCTT
CTATGAATCGACACTAGTTCTAGTAGGTCCCCAGATTAGCCTTTTCAATTTCAGAAAGAATGCTAACCCACAGATGG
TTAGAGAGGCTTACGCAGCAGGTCTCATCAAGACGATCTACCCGAGCAATAATCTCCAGGAAATCAAATACCTTCCC
AAGAAGGTTAAAGATGCAGTCAAAAGATTCAGGACTAACTGCATCAAGAACACAGAGAAAGATATATTTCTCAAGAT
CAGAAGTACTATTCCAGTATGGACGATTCAAGGCTTGCTTCACAAACCAAGGCAAGTAATAGAGATTGGAGTCTCTA
AAAAGGTAGTTCCCACTGAATCAAAGGCCATGGAGTCAAAGATTCAAATAGAGGACCTAACAGAACTCGCCGTAAAG
ACTGGCGAACAGTTCATACAGAGTCTCTTACGACTCAATGACAAGAAGAAAATCTTCGTCAACATGGTGGAGCACGA
CACACTTGTCTACTCCAAAAATATCAAAGATACAGTCTCAGAAGACCAAAGGGCAATTGAGACTTTTCAACAAAGGG
TAATATCCGGAAACCTCCTCGGATTCCATTGCCCAGCTATCTGTCACTTTATTGTGAAGATAGTGGAAAAGGAAGGT
GGCTCCTACAAATGCCATCATTGCGATAAAGGAAAGGCCATCGTTGAAGATGCCTCTGCCGACAGTGGTCCCAAAGA
TGGACCCCCACCCACGAGGAGCATCGTGGAAAAAGAAGACGTTCCAACCACGTCTTCAAAGCAAGTGGATTGATGTG
ATATCTCCACTGACGTAAGGGATGACGCACAATCCCACTATCCTTCGCAAGACCCTTCCTCTATATAAGGAAGTTCA
TTTCATTTGGAGAGAACACGGGGGACTCTAACTAGAGCGGCCGCGAATTCACTAGTGATTGAATTCGGCACGAGGAC
AACATGGCAATTCTAGTCACCGACTTCGTTGTCGCGGCTATAATTTTCTTGATCACTCGGTTCTTAGTTCGTTCTCT
TTTCAAGAAACCAACCCGACCGCTCCCCCCGGGTCCTCTCGGTTGGCCCTTGGTGGGCGCCCTCCCTCTCCTAGGCG
CCATGCCTCACGTCGCACTAGCCAAACTCGCTAAGAAGTATGGTCCGATCATGCACCTAAAAATGGGCACGTGCGAC
ATGGTGGTCGCGTCCACCCCCGAGTCGGCTCGAGCCTTCCTCAAAACGCTAGACCTCAACTTCTCCAACCGCCCACC
CAACGCGGGCGCATCCCACCTAGCGTACGGCGCGCAGGACTTAGTCTTCGCCAAGTACGGTCCGAGGTGGAAGACTT
TAAGAAAATTGAGCAACCTCCACATGCTAGGCGGGAAGGCGTTGGATGATTGGGCAAATGTGAGGGTCACCGAGCTA
GGCCACATGCTTAAAGCCATGTGCGAGGCGAGCCGGTGCGGGGAGCCCGTGGTGCTGGCCGAGATGCTCACGTACGC
CATGGCGAACATGATCGGTCAAGTGATACTCAGCCGGCGCGTGTTCGTGACCAAAGGGACCGAGTCTAACGAGTTCA
AAGACATGGTGGTCGAGTTGATGACGTCCGCCGGGTACTTCAACATCGGTGACTTCATACCCTCGATCGCTTGGATG
GATTTGCAAGGGATCGAGCGAGGGATGAAGAAGCTGCACACGAAGTTTGATGTGTTATTGACGAAGATGGTGAAGGA
GCATAGAGCGACGAGTCATGAGCGCAAAGGGAAGGCAGATTTCCTCGACGTTCTCTTGGAAGAATGCGACAATACAA
ATGGGGAGAAGCTTAGTATTACCAATATCAAAGCTGTCCTTTTGAATCTATTCACGGCGGGCACGGACACATCTTCG
AGCATAATCGAATGGGCGTTAACGGAGATGATCAAGAATCCGACGATCTTAAAAAAGGCGCAAGAGGAGATGGATCG
AGTCATCGGTCGTGATCGGAGGCTGCTCGAATCGGACATATCGAGCCTCCCGTACCTACAAGCCATTGCTAAAGAAA
CGTATCGCAAACACCCGTCGACGCCTCTCAACTTGCCGAGGATTGCGATCCAAGCATGTGAAGTTGATGGCTACTAC
ATCCCTAAGGACGCGAGGCTTAGCGTGAACATTTGGGCGATCGGTCGGGACCCGAATGTTTGGGAGAATCCGTTGGA
GTTCTTGCCGGAAAGATTCTTGTCTGAAGAGAATGGGAAGATCAATCCCGGTGGGAATGATTTTGAGCTGATTCCGT
TTGGAGCCGGGAGGAGAATTTGTGCGGGGACAAGGATGGGAATGGTCCTTGTAAGTTATATTTTGGGCACTTTGGTC
CATTCTTTTGATTGGAAATTACCAAATGGTGTCGCTGAGCTTAATATGGATGAAAGTTTTGGGCTTGCATTGCAAAA
GGCCGTGCCGCTCTCGGCCTTGGTCAGCCCACGGTTGGCCTCAAACGCGTACGCAACCTGAGCTAATGGGCTGGGCC
TAGTTTTGTGGGCAATCGAATTCCCGCGGTGGAGCTCCGGCCATGCTAGAGTCCGCAAAAATCACCAGTCTCTCTCT
ACAAATCTATCTCTCTCTATTTTTCTCCAGAATAATGTGTGAGTAGTTCCCAGATAAGGGAATTAGGGTTCTTATAG
GGTTTCGCTCATGTGTTGAGCATATAAGAAACCCTTAGTATGTATTTGTATTTGTAAAATACTTCTATCAATAAAAT
TTCTAATTCCTAAAACCAAAATCCAGTGACCTCTGCAGGTCCCCAGATTAGCCTTTTCAATTTCAGAAAGAATGCTA
ACCCACAGATGGTTAGAGAGGCTTACGCAGCAGGTCTCATCAAGACGATCTACCCGAGCAATAATCTCCAGGAAATC
AAATACCTTCCCAAGAAGGTTAAAGATGCAGTCAAAAGATTCAGGACTAACTGCATCAAGAACACAGAGAAAGATAT
ATTTCTCAAGATCAGAAGTACTATTCCAGTATGGACGATTCAAGGCTTGCTTCACAAACCAAGGCAAGTAATAGAGA
TTGGAGTCTCTAAAAAGGTAGTTCCCACTGAATCAAAGGCCATGGAGTCAAAGATTCAAATAGAGGACCTAACAGAA
CTCGCCGTAAAGACTGGCGAACAGTTCATACAGAGTCTCTTACGACTCAATGACAAGAAGAAAATCTTCGTCAACAT
GGTGGAGCACGACACACTTGTCTACTCCAAAAATATCAAAGATACAGTCTCAGAAGACCAAAGGGCAATTGAGACTT
TTCAACAAAGGGTAATATCCGGAAACCTCCTCGGATTCCATTGCCCAGCTATCTGTCACTTTATTGTGAAGATAGTG
GAAAAGGAAGGTGGCTCCTACAAATGCCATCATTGCGATAAAGGAAAGGCCATCGTTGAAGATGCCTCTGCCGACAG
TGGTCCCAAAGATGGACCCCCACCCACGAGGAGCATCGTGGAAAAAGAAGACGTTCCAACCACGTCTTCAAAGCAAG
TGGATTGATGTGATATCTCCACTGACGTAAGGGATGACGCACAATCCCACTATCCTTCGCAAGACCCTTCCTCTATA
TAAGGAAGTTCATTTCATTTGGAGAGAACACGGGGGACTCTAGGGGGATCTACC**ATG**AGCCCAGAACGACGCCCGGC
CGACATCCGCCGTGCCACCGAGGCGGACATGCCGGCGGTCTGCACCATCGTCAACCACTACATCGAGACAAGCACGG
TCAACTTCCGTACCGAGCCGCAGGAACCGCAGGAGTGGACGGACGACCTCGTCCGTCTGCGGGAGCGCTATCCCTGG
CTCGTCGCCGAGGTGGACGGCGAGGTCGCCGGCATCGCCTACGCGGGCCCCTGGAAGGCACGCAACGCCTACGACTG
GACGGCCGAGTCGACCGTGTACGTCTCCCCCCGCCACCAGCGGACGGACTGGGCTCCACGCTCTACACCCACCTGC
TGAAGTCCCTGGAGGCACAGGGCTTCAAGAGCGTGGTCGCTGTCATCGGGCTGCCCAACGACCCGAGCGTGCGCATG
CACGAGGCGCTCGGATATGCCCCCCGCGGCATGCTGCGGGCGGCCGGCTTCAAGCACGGGAACTGGCATGACGTGGG

TTTCTGGCAGCTGGACTTCAGCCTGCCGGTACCGCCCCGTCCGGTCCTGCCCGTCACCGAGATC**TGA**TGACCCCGAA
TTTCCCCGATCGTTCAAACATTTGGCAATAAAGTTTCTTAAGATTGAATCCTGTTGCCGGTCTTGCGATGATTATCA
TATAATTTCTGTTGAATTACGTTAAGCATGTAATAATTAACATGTAATGCATGACGTTATTTATGAGATGGGTTTTT
ATGATTAGAGTCCCGCAATTATACATTTAATACGCGATAGAAAACAAAATATAGCGCGCAAACTAGGATAAATTATC
GCGCGCGGTGTCATCTATGTTACTAGATCGG***GAATTC***AATTCGGCGTTAATTCAGTACATTAAAAACGTCCGCAATG
TGTTATTAAGTTGTCTAAGCGTCAATTTGTTTACACCACAATATATCCTGCCACCAGCCAGCCAACAGCTCCCCGAC
CGGCAGCTCGGCACAAAATCACCACTCGATACAGGCAGCCCATCAGTCCGGGACGGCGTCAGCGGGAGAGCCGTTGT
AAGGCGGCAGACTTTGCTCATGTTACCGATGCTATTCGGAAGAACGGCAACTAAGCTGCCGGGTTTGAAACACGGAT
GATCTCGCGGAGGGTAGCATGTTGATT~~GTAACGATGACAGAGCGTT~~GCTGCCTGTGATCAATTCGGGCACGAACCCA
GTGGACATAAGCCTGTTCGGTTCGTAAGCTGTAATGCAAGTAGCGTATGCGCTCACGCAACTGGTCCAGAACCTTGA
CCGAACGCAGCGGTGGTAACGGCGCAGTGGCGGTTTTCATGGCTTGTTATGACTGTTTTTTTGGGGTACAGTCTATG
CCTCGGGCATCCAAGCAGCAAGCGCGTTACGCCGTGGGTCGATGTTTGATGTTATGGAGCAGCaACGATGTTACGCA
GCAGGGCAGTCGCCCTAAAACAAAGTTAAACATCATGGGGGAAGCGGTGATCGCCGAAGTATCGACTCAACTATCAG
AGGTAGTTGGCGTCATCGAGCGCCATCTCGAACCGACGTTGCTGGCCGTACATTTGTACGGCTCCGCAGTGGATGGC
GGCCTGAAGCCACACAGTGATATTGATTTGCTGGTTACGGTGACCGTAAGGCTTGATGAAACAACGCGGCGAGCTTT
GATCAACGACCTTTTGGAAACTTCGGCTTCCCCTGGAGAGAGCGAGATTCTCCGCGCTGTAGAAGTCACCATTGTTG
TGCACGACGACATCATTCCGTGGCGTTATCCAGCTAAGCGCGAACTGCAATTTGGAGAATGGCAGCGCAATGACATT
CTTGCAGGTATCTTCGAGCCAGCCACGATCGACATTGATCTGGCTATCTTGCTGACAAAAGCAAGAGAACATAGCGT
TGCCTTGGTAGGTCCAGCGGCGGAGGAACTCTTTGATCCGGTTCCTGAACAGGATCTATTTGAGGCGCTAAATGAAA
CCTTAACGCTATGGAACTCGCCGCCCGACTGGGCTGGCGATGAGCGAAATGTAGTGCTTACGTTGTCCCGCATTTGG
TACAGCGCAGTAACCGGCAAAATCGCGCCGAAGGATGTCGCTGCCGACTGGGCAATGGAGCGCCTGCCGGCCCAGTA
TCAGCCCGTCATACTTGAAGCTAGACAGGCTTATCTTGGACAAGAAGAAGATCGCTTGGCCTCGCGCGCAGATCAGT
TGGAAGAATTTGTCCACTACGTGAAAGGCGAGATCACCAAGGTAGTCGGCAAATAATGTCTAGCTAGAAATTCGTTC
AAGCCGACGCCGCTTCGCCGGCGTTAACTCAAGCGATTAGATGCACTAAGCACATAATTGCTCACAGCCAAACTATC
AGGTCAAGTCTGCTTTTATTATTTTTAAGCGTGCATAATAAGCCCTACACAAATTGGGAGATATATCATGCATGACC
AAAATCCCTTAACGTGAGTTTTCGTTCCACTGAGCGTCAGACCCCGTAGAAAAGATCAAAGGATCTTCTTGAGATCC
TTTTTTTCTGCGCGTAATCTGCTGCTTGCAAACAAAAAAACCACCGCTACCAGCGGTGGTTTGTTTGCCGGATCAAG
AGCTACCAACTCTTTTTCCGAAGGTAACTGGCTTCAGCAGAGCGCAGATACCAAATACTGTCCTTCTAGTGTAGCCG
TAGTTAGGCCACCACTTCAAGAACTCTGTAGCACCGCCTACATACCTCGCTCTGCTAATCCTGTTACCAGTGGCTGC
TGCCAGTGGCGATAAGTCGTGTCTTACCGGGTTGGACTCAAGACGATAGTTACCGGATAAGGCGCAGCGGTCGGGCT
GAACGGGGGGTTCGTGCACACAGCCCAGCTTGGAGCGAACGACCTACACCGAACTGAGATACCTACAGCGTGAGCTA
TGAGAAAGCGCCACGCTTCCCGAAGGGAGAAAGGCGGACAGGTATCCGGTAAGCGGCAGGGTCGGAACAGGAGAGCG
CACGAGGGAGCTTCCAGGGGGAAACGCCTGGTATCTTTATAGTCCTGTCGGGTTTCGCCACCTCTGACTTGAGCGTC
GATTTTTGTGATGCTCGTCAGGGGGGCGGAGCCTATGGAAAAACGCCAGCAACGCGGCCTTTTTACGGTTCCTGGCC
TTTTGCTGGCCTTTTGCTCACATGTTCTTTCCTGCGTTATCCCCTGATTCTGTGGATAACCGTATTACCGCCTTTGA
GTGAGCTGATACCGCTCGCCGCAGCCGAACGACCGAGCGCAGCGAGTCAGTGAGCGAGGAAGCGGAAGAGCGCCTGA
TGCGGTATTTTCTCCTTACGCATCTGTGCGGTATTTCACACCGCATATGGTGCACTCTCAGTACAATCTGCTCTGAT
GCCGCATAGTTAAGCCAGTATACACTCCGCTATCGCTACGTGACTGGGTCATGGCTGCGCCCCGACACCCGCCAACA
CCCGCTGACGCGCCCTGACGGGCTTGTCTGCTCCCGGCATCCGCTTACAGACAAGCTGTGACCGTCTCCGGGAGCTG
CATGTGTCAGAGGTTTTCACCGTCATCACCGAAACGCGCGAGGCAGGGTGCCTTGATGTGGGCGCCGGCGGTCGAGT
GGCGACGGCGCGGCTTGTCCGCGCCCTGGTAGATTGCCTGGCCGTAGGCCAGCCATTTTTGAGCGGCCAGCGGCCGC
GATAGGCCGACGCGAAGCGGCGGGGCGTAGGGAGCGCAGCGACCGAAGGGTAGGCGCTTTTTGCAGCTCTTCGGCTG
TGCGCTGGCCAGACAGTTATGCACAGGCCAGGCGGGTTTTAAGAGTTTTAATAAGTTTTAAAGAGTTTTAGGCGGAA
AAATCGCCTTTTTTCTCTTTTATATCAGTCACTTACATGTGTGACCGGTTCCCAATGTACGGCTTTGGGTTCCCAAT
GTACGGGTTCCGGTTCCCAATGTACGGCTTTGGGTTCCCAATGTACGTGCTATCCACAGGAAAGAGACCTTTTCGAC
CTTTTTCCCCTGCTAGGGCAATTTGCCCTAGCATCTGCTCCGTACATTAGGAACCGGCGGATGCTTCGCCCTCGATC
AGGTTGCGGTAGCGCATGACTAGGATCGGGCCAGCCTGCCCCGCCTCCTCCTTCAAATCGTACTCCGGCAGGTCATT
TGACCCGATCAGCTTGCGCACGGTGAAACAGAACTTCTTGAACTCTCCGGCGCTGCCACTGCGTTCGTAGATCGTCT
TGAACAACCATCTGGCTTCTGCCTTGCCTGCGGCGCGGCGTGCCAGGCGGTAGAGAAAACGGCCGATGCCGGGATCG
ATCAAAAAGTAATCGGGGTGAACCGTCAGCACGTCCGGGTTCTTGCCTTCTGTGATCTCGCGGTACATCCAATCAGC
TAGCTCGATCTCGATGTACTCCGGCCGCCCGGTTTCGCTCTTTACGATCTTGTAGCGGCTAATCAAGGCTTCACCCT
CGGATACCGTCACCAGGCGGCCGTTCTTGGCCTTCTTCGTACGCTGCATGGCAACGTGCGTGGTGTTTAACCGAATG
CAGGTTTCTACCAGGTCGTCTTTCTGCTTTCCGCCATCGGCTCGCCGGCAGAACTTGAGTACGTCCGCAACGTGTGG
ACGGAACACGCGGCCGGGCTTGTCTCCCTTCCCTTCCCGGTATCGGTTCATGGATTCGGTTAGATGGGAAACCGCCA
TCAGTACCAGGTCGTAATCCCACACACTGGCCATGCCGGCCGGCCCTGCGGAAACCTCTACGTGCCCGTCTGGAAGC
TCGTAGCGGATCACCTCGCCAGCTCGTCGGTCACGCTTCGACAGACGGAAAACGGCCACGTCCATGATGCTGCGACT

ATCGCGGGTGCCCACGTCATAGAGCATCGGAACGAAAAAATCTGGTTGCTCGTCGCCCTTGGGCGGCTTCCTAATCG
ACGGCGCACCGGCTGCCGGCGGTTGCCGGGATTCTTTGCGGATTCGATCAGCGGCCGCTTGCCACGATTCACCGGGG
CGTGCTTCTGCCTCGATGCGTTGCCGCTGGGCGGCCTGCGCGGCCTTCAACTTCTCCACCAGGTCATCACCCAGCGC
CGCGCCGATTTGTACCGGGCCGGATGGTTTGCGACCGTCACGCCGATTCCTCGGGCTTGGGGGTTCCAGTGCCATTG
CAGGGCCGGCAGACAACCCAGCCGCTTACGCCTGGCCAACCGCCCGTTCCTCCACACATGGGGCATTCCACGGCGTC
GGTGCCTGGTTGTTCTTGATTTTCCATGCCGCCTCCTTTAGCCGCTAAAATTCATCTACTCATTTATTCATTTGCTC
ATTTACTCTGGTAGCTGCGCGATGTATTCAGATAGCAGCTCGGTAATGGTCTTGCCTTGGCGTACCGCGTACATCTT
CAGCTTGGTGTGATCCTCCGCCGGCAACTGAAAGTTGACCCGCTTCATGGCTGGCGTGTCTGCCAGGCTGGCCAACG
TTGCAGCCTTGCTGCTGCGTGCGCTCGGACGGCCGGCACTTAGCGTGTTTGTGCTTTTGCTCATTTTCTCTTTACCT
CATTAACTCAAATGAGTTTTGATTTAATTTCAGCGGCCAGCGCCTGGACCTCGCGGGCAGCGTCGCCCTCGGGTTCT
GATTCAAGAACGGTTGTGCCGGCGGCGGCAGTGCCTGGGTAGCTCACGCGCTGCGTGATACGGGACTCAAGAATGGG
CAGCTCGTACCCGGCCAGCGCCTCGGCAACCTCACCGCCGATGCGCGTGCCTTTGATCGCCCGCGACACGACAAAGG
CCGCTTGTAGCCTTCCATCCGTGACCTCAATGCGCTGCTTAACCAGCTCCACCAGGTCGGCGGTGGCCCATATGTCG
TAAGGGCTTGGCTGCACCGGAATCAGCACGAAGTCGGCTGCCTTGATCGCGGACACAGCCAAGTCCGCCGCCTGGGG
CGCTCCGTCGATCACTACGAAGTCGCGCCGGCCGATGGCCTTCACGTCGCGGTCAATCGTCGGGCGGTCGATGCCGA
CAACGGTTAGCGGTTGATCTTCCCGCACGGCCGCCCAATCGCGGGCACTGCCCTGGGGATCGGAATCGACTAACAGA
ACATCGGCCCCGGCGAGTTGCAGGGCGCGGGCTAGATGGGTTGCGATGGTCGTCTTGCCTGACCCGCCTTTCTGGTT
AAGTACAGCGATAACCTTCATGCGTTCCCCTTGCGTATTTGTTTATTTACTCATCGCATCATATACGCAGCGACCGC
ATGACGCAAGCTGTTTTACTCAAATACACATCACCTTTTTAGACGGCGGCGCTCGGTTTCTTCAGCGGCCAAGCTGG
CCGGCCAGGCCGCCAGCTTGGCATCAGACAAACCGGCCAGGATTTCATGCAGCCGCACGGTTGAGACGTGCGCGGGC
GGCTCGAACACGTACCCGGCCGCGATCATCTCCGCCTCGATCTCTTCGGTAATGAAAAACGGTTCGTCCTGGCCGTC
CTGGTGCGGTTTCATGCTTGTTCCTCTTGGCGTTCATTCTCGGCGGCCGCCAGGGCGTCGGCCTCGGTCAATGCGTC
CTCACGGAAGGCACCGCGCCGCCTGGCCTCGGTGGGCGTCACTTCCTCGCTGCGCTCAAGTGCGCGGTACAGGGTCG
AGCGATGCACGCCAAGCAGTGCAGCCGCCTCTTTCACGGTGCGGCCTTCCTGGTCGATCAGCTCGCGGGCGTGCGCG
ATCTGTGCCGGGGTGAGGGTAGGGCGGGGGCCAAACTTCACGCCTCGGGCCTTGGCGGCCTCGCGCCCGCTCCGGGT
GCGGTCGATGATTAGGGAACGCTCGAACTCGGCAATGCCGGCGAACACGGTCAACACCATGCGGCCGGCCGGCGTGG
TGGTGTCGGCCCACGGCTCTGCCAGGCTACGCAGGCCCGCGCCGGCCTCCTGGATGCGCTCGGCAATGTCCAGTAGG
TCGCGGGTGCTGCGGGCCAGGCGGTCTAGCCTGGTCACTGTCACAACGTCGCCAGGGCGTAGGTGGTCAAGCATCCT
GGCCAGCTCCGGGCGGTCGCGCCTGGTGCCGGTGATCTTCTCGGAAAACAGCTTGGTGCAGCCGGC

**Figure S2.** Complete plasmid sequences and corresponding vector maps used for transformation.

**Table S1.** Primer sequences used for amplification and transgene detection.

| Size(bp) | Name | Sequence |
|---|---|---|
| 609 | Vt-F35H_5F | TGACCAAAGGGACCGAGTCT |
| | Vt-F35H_5R | TCGCCCAAATGTTCACGCTA |
| 743 | Rh_NHX_3F | AATCGGTGGGTGAACGAGTC |
| | Rh_NHX_5R | GCCAGGTGTAATGGGACATCA |
| 363 | CPS-F | GTTCAATGGAGGAGTCCCTGT |
| | CPS-R | TCCGGTAATGCCAACTGACT |
| 426 | KS-F | GATGTCTCCGCAGATCGGTT |
| | KS-R | CCGATGTATCGATTGGCACG |
| 404 | KO-F | CCCAACTGCCGTACCTGAAT |
| | KO-R | TTTGTGAGTGGTGAGCCCAA |
| 431 | KAO-F | CCGCTGTGGAACTGATTGGA |
| | KAO-R | CAAGAAATTCCCGGCCCTCT |
| 368 | GA20ox1-F | GCGGACCAAAGCGAGTCCA |
| | GA20ox1-R | AAGGCGTTCAAATTCGGGCT |
| 596 | GA3ox-F | GCGGATGGCATTTCTGGTTAC |
| | GA3ox-R | GGCGAGATTTGGACACTAGC |
| 488 | GA2ox-F | TCCGTTCTGCTGTTGAGGAC |
| | GA2ox-R | TGCCATTACTGACGCCAGAG |
| 378 | GA2ox6-F | AGGGTTGAAGAGCCATGACG |
| | GA2ox6-R | ACCCCAGCAAGCACTTTTGA |
| 116 | RhACT1_F | GTTCCCAGGAATCGCTGATA |
| | RhACT1_R | ATCCTCCGATCCAAACACTG |
| 316 | RhACT2_F | TCCCGCTATGTATGTTGCCAT |
| | RhACT2_R | AGAGCTGGTTTTGGCAGTTT |
